# Differential Gene Expression in Primary Cultured Sensory and Motor Nerve Fibroblasts

**DOI:** 10.3389/fnins.2018.01016

**Published:** 2019-01-09

**Authors:** Qianru He, Mi Shen, Fang Tong, Meng Cong, Shibo Zhang, Yanpei Gong, Fei Ding

**Affiliations:** ^1^Key Laboratory of Neuroregeneration of Jiangsu and Ministry of Education, Co-innovation Center of Neuroregeneration, Jiangsu Clinical Medicine Center of Tissue Engineering and Nerve Injury Repair, Nantong University, Nantong, China; ^2^Department of Hand Surgery, Affiliated Hospital of Nantong University, Nantong, China

**Keywords:** peripheral nervous system, sensory fibroblasts, motor fibroblasts, cell proliferation, cell migration

## Abstract

Fibroblasts (Fbs) effectively promote Schwann cells (SCs) migration, proliferation, and neurite regeneration. Whether Fbs express different motor and sensory phenotypes that regulate the cell behavior and peripheral nerve function has not been elucidated. The present study utilized the whole rat genome microarray analysis and identified a total of 121 differentially expressed genes between the primary cultured motor and sensory Fbs. The genes with high expression in sensory Fbs were related to proliferation, migration, chemotaxis, motility activation, protein maturation, defense response, immune system, taxis, and regionalization, while those with high expression in motor Fbs were related to neuron differentiation, segmentation, and pattern specification. Thus, the significant difference in the expression of some key genes was found to be associated with cell migration and proliferation, which was further validated by quantitative real-time PCR (qPCR). The cell proliferation or migration analysis revealed a higher rate of cell migration and proliferation of sensory Fbs than motor Fbs. Moreover, the downregulated expression of chemokine (C-X-C motif) ligand 10 (CXCL10) and chemokine (C-X-C motif) ligand 3 (CXCL3) suppressed the proliferation rate of sensory Fbs, while it enhanced that of the motor Fbs. However, the migration rate of both Fbs was suppressed by the downregulated expression of CXCL10 or CXCL3. Furthermore, a higher proportion of motor or sensory SCs migrated toward their respective (motor or sensory) Fbs; however, few motor or sensory SCs co-cultured with the other type of Fbs (sensory or motor, respectively), migrated toward the Fbs. The current findings indicated that Fbs expressed the distinct motor and sensory phenotypes involved in different patterns of gene expression, biological processes, and effects on SCs. Thus, this study would provide insights into the biological differences between motor and sensory Fbs, including the role in peripheral nerve regeneration.

## Introduction

A peripheral nerve fiber is mainly composed of neuronal axons, Schwann cells (SCs), and fibroblasts (Fbs). Each axon is wrapped by SCs and surrounded by a layer of connective tissue known as the endoneurium. The axons are bundled together into groups called fascicles, and each fascicle is wrapped in a layer of connective tissue termed as the perineurium. Finally, the whole nerve is wrapped in a layer of connective tissue, known as the epineurium. Fbs are critical components of all nerve compartments, including endoneurium, perineurium, and epineurium (Dreesmann et al., 2009). SCs are shown to play a critical role in the regeneration process after peripheral nerve injury (Soares et al., 2005), whereas Fbs are deemed to impede the axonal regrowth. After peripheral nerve injury, collagen secreted by Fbs can form a scar and prevent nerve regeneration (Atkins et al., 2006). This theory of Fbs preventing the axonal regeneration was challenged by the following evidence, which showed that Fbs provide an environment conducive to axonal regeneration rather than inhibition. The evidence showed that cultured sciatic nerve Fbs, including motor and sensory Fbs, release soluble pro-migratory factors, such as neuregulin-1β1 to promote the migration of SCs (Dreesmann et al., 2009). In addition, the study indicated that perineurial Fbs regulated cell sorting via ephrin-B/EphB2 signaling pathway in SCs and Fbs after peripheral nerve transection, followed by directional collective migration of SCs required to guide the axon regrowth across the wound (Parrinello et al., 2010). A recent study indicated that epineurial Fb-conditioned media promoted SCs migration and outgrowth of neurites in the primary cultured dorsal root ganglion neurons (van Neerven et al., 2013). Tenascin-C protein secreted by the peripheral nerve Fbs enhanced the migration of SCs through the β1-integrin signaling pathway during nerve regeneration (Zhang Z. et al., 2016). Moreover, the study also showed that co-transplanting the sciatic nerve Fbs with SCs at a ratio of 1:2 improved the regeneration and contributed to significantly satisfactory functional recovery at 3 months post-sciatic nerve transection (Wang et al., 2017). These findings demonstrated that Fbs improved the regeneration by modulating both SC behavior (i.e., migration and proliferation) and neurites outgrowth; for example, sprouting and directional growth. However, the Fbs used in the above studies were derived from the sciatic nerve, which includes motor and sensory nerves. In the peripheral nervous system, motor nerves (efferent nerves) conduct signals from the central nervous system (CNS) to the muscles, while the sensory nerves (afferent nerves) conduct sensory information from their receptors to the CNS. The different phenotypes of Fbs in motor and sensory nerves are yet to be elucidated.

The study utilized proteomics to detect the protein profile of the peripheral motor and sensory nerves. Consequently, we found 100 proteins that exhibited converse expression patterns between the motor and sensory nerves. These proteins are expressed in different cell types, including neurons, SCs, and Fbs (He et al., 2012b). Thus, exploring the different motor and sensory neural pathways would contribute to the knowledge of the complicated molecular mechanisms underlying peripheral nerve development and regeneration. Furthermore, SCs support the peripheral nerve regeneration by expressing different motor and sensory phenotypes and neurotrophic factors (Hoke et al., 2006; Brushart et al., 2013). However, whether peripheral nerve Fbs also express distinct motor and sensory phenotypes to regulate cell behavior and nerve regeneration is yet to be clarified.

The present study aimed to compare the gene expression of the motor and sensory Fbs using the whole rat genome microarray analysis, thereby contributing to our understanding of the distinct functions between both Fbs in the sensory and motor nerves.

## Materials and Methods

### Primary Cell Culture and Immunocytochemistry

This study was carried out in accordance with the Institutional Animal Care Guidelines of Nantong University. All animal procedures were ethically approved by the Administration Committee of Experimental Animals, Jiangsu Province, China. Sprague–Dawley (SD) rats (7-days-old) were provided by the Experimental Animal Center of Nantong University of China. Both Fbs and SCs were derived from P7 rat and cultured as described previously (Mathon et al., 2001; Parrinello et al., 2010).

In order to obtain motor and sensory nerve Fbs and SCs, SD rats were sanitized using 70% ethanol prior to decapitation. The vertebral canal was carefully opened to expose the spinal cord. Then, the ventral root (motor nerve) and dorsal root (sensory nerve) were excised, from which, motor and sensory nerves were harvested, respectively and placed in ice-cold D-Hank’s solution. The nerves were sliced into 3–5 mm pieces and digested with 0.125% (w/v) trypsin for 15–20 min, followed by culture in DMEM supplemented with 10% fetal bovine serum (FBS) (Gibco, Grand Island, NY, United States) for 4 days at 37°C in the presence of 5% CO_2_. The cells were subjected to differential digestion and adhesion sequentially to remove the SCs as described previously (Parrinello et al., 2010; He et al., 2012a). These excluded SCs were utilized for the co-culture experiment.

After the purification and proliferation of motor and sensory Fbs, the cells were stained with anti-Thy-1 (a specific marker for Fb) by immunocytochemistry. Briefly, motor and sensory Fbs were washed with phosphate-buffered saline (PBS, pH 7.2) and fixed in 4% paraformaldehyde (pH 7.4) for 20 min at room temperature. The samples were permeabilized using 0.3% Triton X-100 in 0.01 M PBS containing 10% goat serum for 60 min at 37°C, followed by incubation with the mouse anti-Thy-1 antibody (1:400, Abcam, Cambridge, MA, United States) at 4°C overnight, and then, TRITC-labeled goat anti-mouse IgG (1:400, Santa Cruz Biotechnology, Santa Cruz, CA, United States) for 2 h. Then, the samples were stained with 5 μg/mL Hoechst 33342 dye for 10 min at 37°C and images captured using a confocal laser scanning microscope (TCS SP5, Leica Microsystems, Germany).

### Flow Cytometry Analysis (FCA)

The purity of motor and sensory Fbs was evaluated by FCA as described previously (He et al., 2012a). The motor and sensory Fbs were digested by trypsin, and the cell pellets were resuspended and incubated in fixation medium (Invitrogen, Carlsbad, CA, United States) at room temperature for 15 min. Next, the cells were incubated with the permeabilization medium (Invitrogen) and probed with an anti-mouse anti-Thy-1 antibody (Becton Dickinson Biosciences, San Jose CA, United States) simultaneously for 20 min. Subsequently, the cells were incubated with the TRITC-labeled goat anti-mouse IgG (1:200; Santa Cruz Biotechnology) for 30 min. Flow cytometry was performed by FACSCalibur, and the data analyzed by CellQuest software (Becton Dickinson Biosciences, San Jose CA, United States). The cells incubated only with TRITC-labeled secondary antibody served as a negative control.

### Whole Rat Genome Microarray Analysis

The cultured motor and sensory Fbs were harvested, and the samples were subjected to the whole rat genome microarray analysis by Shanghai Biotechnology Co., Ltd (Shanghai, China). Total RNA from the motor and sensory Fbs was extracted using TRIzol reagent (Cat# 15596-018, Life Technologies, Carlsbad, CA, United States) according to the manufacturer’s instructions, the quality of the RNA was examined using an Agilent Bioanalyzer 2100 (Agilent Technologies, Santa Clara, CA, United States). The total RNA was further purified using the RNeasy Micro Kit (Cat# 74004, Qiagen, GmBH, Germany) and RNase-Free DNase Set (Cat# 79254, Qiagen) according to the manufacturer’s protocol. The RNA samples were quantitated using NanoDrop ND-1000 UV-VIS Spectrophotometer. Next, total RNA was amplified and labeled using the Low Input Quick Amp Labeling Kit, One-Color (Cat# 5190-2305, Agilent Technologies), following the manufacturer’s instructions. The labeled cRNA was purified by RNeasy Mini Kit (Ca# 74106, Qiagen).

Hybridization experiments were performed using Agilent Whole Rat Genome Oligo Microarray (4 × 44K) (design ID: 014879, Agilent Technologies). Each slide was hybridized with 1.65 μg Cy3-labeled cRNA using Gene Expression Hybridization Kit (Cat# 5188-5242, Agilent technologies) in a hybridization oven (Cat# G2545A, Agilent Technologies), according to the manufacturer’s instructions. After 17 h, the slides were washed in staining dishes (Cat# 121, Thermo Shandon, Waltham, MA, United States) with Gene Expression Wash Buffer Kit (Cat# 5188-5327, Agilent Technologies) and scanned by Agilent Microarray Scanner (Cat# G2565CA, Agilent Technologies) using default settings as follows: Dye channel: Green, Scan resolution = 5 μm, PMT 100%, 10%, 16 bit. Raw data were extracted with Feature Extraction Software 10.7 (Agilent Technologies) and normalized by Quantile algorithm, Gene Spring Software 11.0 (Agilent technologies).

Four replicates were analyzed for each sample category for a total of eight arrays. The differentially expressed genes were identified by >2-fold difference in gene expression and a *P*-value < 0.05 (Clarke et al., 2008).

### Quantitative Real-Time PCR

Total RNA was extracted from the motor and sensory Fbs using TRIzol (Sigma), and cDNA was synthesized according to the instructions of the Omniscript^®^ RT Kit (Qiagen, Valencia, CA, United States). qPCR was performed in CFX96^TM^ Real-Time PCR System (Bio-Rad, Singapore) using FastStart^®^ SYBR Green qPCR Master Mix (Roche, Mannheim, Germany) according to the specifications. The reaction mixture consisted of 2 μL cDNA from each sample, 1 μL each primer, 6 μL RNase/DNase-free water, and 10 μL SYBR Green qPCR Master Mix. The three-step fast cycle protocol was applied. After amplification, the melting curves were analyzed. PCR products were quantified with the 2^-ΔCT^. The primers (Supplementary Table S1) used in qPCR were synthesized by the Shanghai Generay Biotech Co., Ltd, China.

### Real-Time Cell Analyzer (RTCA)

Real-time cell analyzer (xCELLigence, Roche, San Diego, CA, United States) monitors the live cell proliferation, migration, viability, and morphology examined by label-free assays (Ke et al., 2011). Briefly, a volume of 50 μL cell culture medium was added to E-plates and the background intensity measured. E-plates consists of incorporated sensor electrode arrays such that cells inside each well can be monitored and assayed. In order to evaluate the cell proliferation capacity, an equivalent of 5,000 (5K), 10K and 20K motor and sensory Fbs were resuspended in 100 μL DMEM supplemented with 10% FBS and seeded into each well. Immediately, the E-plate was installed into the RTCA system, and the cell index (a quantitative measure of cell number) was measured every 15 min. Similarly, the cell migration of the sensory and motor Fbs was also analyzed by the RTCA method. Briefly, a volume of 165 μL DMEM supplemented with 10% FBS was added to the bottom chamber of the CIM plates (cell invasion and migration plate), while a volume of 165 μL serum-free DMEM was added to the bottom chamber as control, then 50 μL of serum-free DMEM was added to the top chamber of the plates to measure the background intensity. Furthermore, to evaluate the migration capacity, 20000 and 40000 Fbs were resuspended in 100 μL serum-free DMEM and seeded into the top chamber of CIM plates for measuring the cell index every 15 min.

### Cell Proliferation Assay

The proliferation capacity of the motor and sensory Fbs was evaluated through the 5-ethynyl-2′-deoxyuridine (EdU) Labeling Kit (Ribobio, Guangzhou, China). EdU is a thymidine analog, which is incorporated into the DNA of dividing cells and is widely used to track the proliferating cells in multiple biological systems. The cultured Fbs are mixed with 50 μm EdU. Also, the fixed cells were stained using anti-EdU solution to allow the EdU incorporation into RNA and Hoechst 33342 dye to stain the cell nucleus. Subsequently, the cells were imaged under a Nikon fluorescence microscope in five random fields for each well. The total number of nuclei and EdU-positive nuclei was enumerated and the percentage of EdU-positive nuclei calculated.

### Transwell Migration Assay

Transwell migration assay has been widely employed for studying the motility of different types of cells. The migration of motor and sensory Fbs was assessed in 6.5-mm transwell plate with 8 μm pores (Costar, Corning Inc., Corning, NY, United States) as described previously (Nguyen et al., 1999). Briefly, DMEM supplemented with 10% FBS was added to the bottom of a transwell-plate, and the motor and sensory Fbs (5 × 10^5^) in serum-free DMEM were seeded into the upper chamber of the plate. The assembly was incubated at 37°C (5% CO_2_) for 4 or 24 h to allow the migration of the cells. The Fbs on the upper surface of the insert membranes were cleaned by cotton swabs, and those that passed through the membrane were stained by crystal violet for 30 min. The number of motor and sensory Fbs on the bottom of the insert membrane was counted in four random fields for each well.

### siRNA Transfection

For the transfection of Fbs, siRNAs (1, 2, and 3) for CXCL3, CXCL10 (1, 2, and 3), and non-targeting negative control (NTC) were synthesized by Ribobio. Lipofectamine 2000 transfection reagent (Invitrogen) and Opti-MEM I reduced serum medium (Invitrogen) were used for the transfection experiments according to the manufacturer’s specifications. The sequences of siRNA duplexes are listed in Supplementary Table S2.

### Western Blot Assay

The protein from the motor and sensory Fbs was extracted using the M-PER mammalian protein extraction reagent (Pierce, Rockford, IL, United States) and estimated with the BCA protein assay kit (Pierce). An equivalent of 30 μg of total protein was resolved by 10% (w/v) SDS-PAGE and transferred to the PVDF membrane (Millipore, Bedford, MA, United States). The membranes were blocked with 5% (w/v) non-fat dry milk in TBS-T (0.05% (v/v) (Tween 20 in Tris-buffered saline) for 1 h and probed overnight at 4°C overnight with the following primary antibodies: rabbit polyclonal anti-CXCL10 (1:1000; Abcam), rabbit monoclonal anti-CXCL3 (1:1000; Abcam), and rabbit polyclonal anti-β-actin (1:10000; Abcam). Subsequently, the membrane was incubated with IRDye 800-conjugated secondary antibodies (Odyssey, Lincoln, NE, United States) for 1 h at room temperature. The Odyssey infrared image system was used to scan the images, and intensity of the immunoreactive bands was analyzed by NIH ImageJ software. β-Actin was used as the internal reference.

### Co-culture Assay

For the co-culture of Fbs and SCs, 5 × 10^5^ motor and sensory Fbs were seeded in DMEM, supplemented with 10% FBS in the bottom chamber of the transwell plate, and cultured for 24 h. 8 × 10^5^ motor and sensory SCs in DMEM were seeded in the upper chamber of the transwell-plate for co-culture for 12 h. The SCs on the upper surface of the insert membranes were cleaned by cotton swabs, and those that passed through the membrane were stained with for crystal violet for 30 min. The number of motor and sensory SCs on the bottom of the insert membrane was counted in five random fields for each well. The co-culture experiment was conducted on six groups: motor or sensory SCs without Fbs as control, motor Fbs + motor SCs, motor Fbs + sensory SCs, sensory Fbs + motor SCs, sensory Fbs + sensory SCs.

### Statistical Analysis

All data were expressed as the means ± SEM. Statistical differences in the data were evaluated by unpaired *t*-test and one-way ANOVA using GraphPad Prism 6.0. The criterion for statistical significance was set at *p* < 0.05. All assays were performed in triplicate unless otherwise specified.

## Results

### Evaluation of Purity of Sensory and Motor Fibroblasts

**FIGURE 1:**
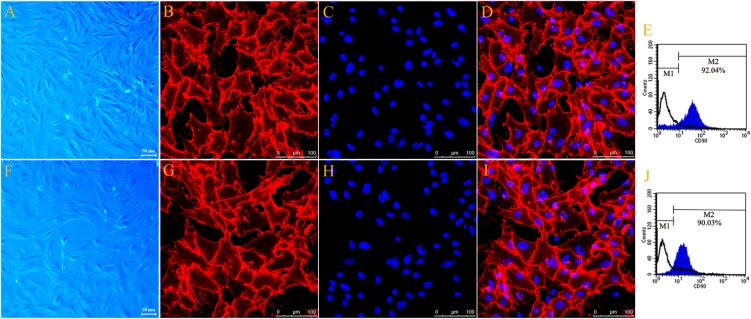
Phase-contrast micrograph showed typical cell morphology of primary cultured motor **(A)** and sensory Fbs **(F)** (scale bar, 50 μm). Fluorescence microscope photograph (scale bar, 100 μm) of cultured motor **(B–D)** and sensory fibroblasts **(G–I)** showing immunostaining with antibody against Thy-1 (**B,G**, green), Hoechst 33342 nucleus staining (**C,H**, blue), and the merge of both staining **(D,I)**, *n* = 9 rats. The FCA graph showing the percentage of Thy-1-positive cells (M2) of cultured motor **(E)** and sensory **(J)** fibroblasts, *n* = 10 rats.

The typical cell morphology of the primary cultured motor and sensory Fbs is shown using phase-contrast micrograph (Figures 1A,F). The motor and sensory Fbs were labeled using anti-Thy-1 and visualized using a confocal laser scanning microscope (Figures 1B,G), respectively. Hoechst 33342 dye was used to label the cell nucleus (Figures 1C,H). Figures 1D,I show the merged images of Fb immunostaining and nuclear staining indicating > 90% Thy-1 and Hoechst co-labeled cells. The cell cultures used in the current experiments included primary motor and sensory Fbs, respectively, as indicated using flow cytometry (Figures 1E,J). Furthermore, >90% of all the primary cells in the motor and sensory Fb culture were Fbs, as indicated by M2, while the remaining < 10% (indicated by M1) were SCs. These high purity motor and sensory Fb cultures were used in the subsequent experiments.

### Genome Microarray Analysis of Sensory and Motor Fbs

**FIGURE 2:**
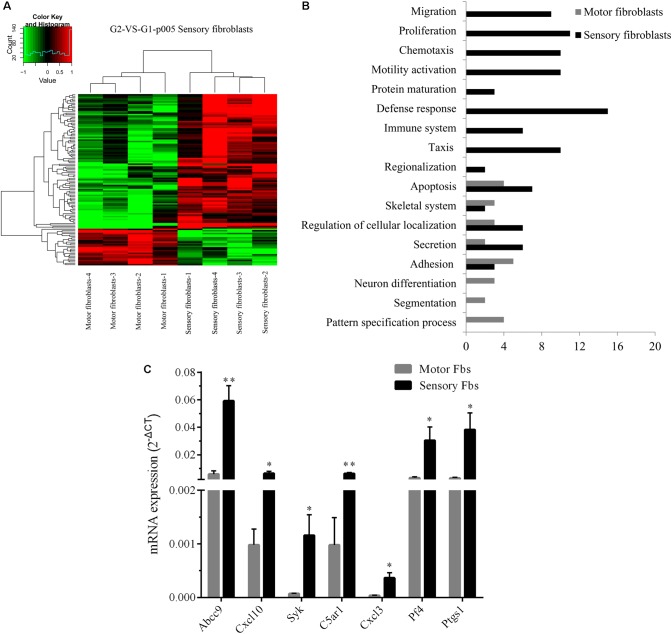
**(A)** Heatmap and cluster dendrogram of the 121 differentially expressed genes that had more than twofold changes (sensory fibroblasts/motor fibroblasts) in their expression of four replicates, *n* = 12 rats. **(B)** Histogram showing functional categorization of the 121 differentially expressed genes between motor and sensory fibroblasts according to their main biological functions collected from DAVID Bioinformatic Resources 6.7 (Rat) and Gene Ontology, in which the different functional categories of the higher expressed genes are shown on the ordinate, and the number of the higher expressed genes within each categories of sensory or motor fibroblasts is shown on the abscissa. **(C)** The qPCR data showing that *Abcc9, Cxcl10, Cxcl3, Syk, C5ar1, Pf4, Ptgs1*, presented the higher mRNA level in sensory fibroblasts than in motor fibroblasts. Data are expressed as means ± SEM of three independent experiments (each in triplicate). GAPDH served as an internal control. *p* = 0.009, *p* = 0.031, *p* = 0.047, *p* = 0.003, *p* = 0.027, *p* = 0.049, and *p* = 0.045, respectively, ^∗^*p* < 0.05, ^∗∗^*p* < 0.01 vs. motor Fbs, unpaired *t*-test, *n* = 9–10 rats.

Whole rat genome microarray was used to analyze the differentially expressed genes in primary cultured sensory and motor Fbs (the original normalized data is listed in Supplementary Table S3) and to identify 121 differentially expressed genes between both Fbs (Supplementary Table S4). A total of 95 genes were significantly upregulated in sensory Fbs compared to motor Fbs, and 26 genes were significantly upregulated in motor Fbs compared to sensory Fbs (Figure 2A). In order to understand the biological significance of genes identified by genome microarray analysis, the differentially expressed genes between the motor and sensory Fbs were categorized based on the biological functions predicted by the DAVID Bioinformatic Resources 6.7 (Rat) and Gene Ontology. The upregulated 95 genes in sensory Fbs were involved in fourteen biological processes: proliferation, migration, motility activation, chemotaxis, adhesion, immune response, apoptosis, secretion, taxis, skeletal system, regionalization, regulation of cellular localization, defense response, and protein maturation (Table 1). The upregulated 26 genes in motor Fbs were involved in nine biological processes: adhesion, neuron differentiation, secretion, skeletal system, apoptosis, embryonic development, segmentation, regionalization, and pattern specification process (Table 2 and Figure 2B).

**Table 1:** Functional classification of upregulated genes in sensory fibroblasts.

Genbank accession	Gene symbol	Gene name	Fold change	*P*-values
		**Proliferation**		
NM_013028	Shox2	Short stature homeobox 2	3.3	0.022
XM_578417	Rerg	RAS-like, estrogen-regulated, growth-inhibitor	2.9	0.001
NM_139089	Cxcl10	Chemokine (C-X-C motif) ligand 10	2.8	0.006
NM_017043	Ptgs1	Prostaglandin-endoperoxide synthase 1	2.6	0.027
NM_030871	Pde1a	Phosphodiesterase 1A, calmodulin-dependent	2.2	0.014
NM_001033998	Itgal	Integrin, alpha L	2.1	0.014
NM_031512	Il1b	Interleukin 1 beta	2.1	0.040
NM_012758	Syk	Spleen tyrosine kinase	2.1	0.032
NM_022634	Lst1	Leukocyte specific transcript 1	2.1	0.011
NM_001107031	Slfn2	Schlafen 2	2.1	0.005
		**Migration**		
NM_013040	Abcc9	ATP-binding cassette, subfamily C (CFTR/MRP), member 9	2.7	0.024
NM_001007729	Pf4	Platelet factor 4	2.7	0.004
NM_138522	Cxcl3	Chemokine (C-X-C motif) ligand 3	2.4	0.019
BC079460	Ccl6	Chemokine (C-C motif) ligand 6	2.2	0.002
NM_031512	Il1b	Interleukin 1 beta	2.1	0.040
NM_012758	Syk	Spleen tyrosine kinase	2.1	0.032
NM_053619	C5ar1	Complement component 5a receptor 1	2.1	0.012
NM_053647	Cxcl2	Chemokine (C-X-C motif) ligand 2	2.1	0.046
		**Motility activation**		
NM_139089	Cxcl10	chemokine (C-X-C motif) ligand 10	2.8	0.006
NM_013040	Abcc9	ATP-binding cassette, subfamily C (CFTR/MRP), member 9	2.7	0.024
NM_001007729	Pf4	Platelet factor 4	2.7	0.004
NM_138522	Cxcl3	Chemokine (C-X-C motif) ligand 3	2.4	0.019
BC079460	Ccl6	Chemokine (C-C motif) ligand 6	2.2	0.002
NM_031512	Il1b	Interleukin 1 beta	2.1	0.040
NM_012758	Syk	Spleen tyrosine kinase	2.1	0.032
NM_053619	C5ar1	Complement component 5a receptor 1	2.1	0.012
NM_053647	Cxcl2	Chemokine (C-X-C motif) ligand 2	2.1	0.046
		**Chemotaxis**		
NM_139089	Cxcl10	Chemokine (C-X-C motif) ligand 10	2.8	0.006
NM_001007729	Pf4	Platelet factor 4	2.7	0.004
NM_138522	Cxcl3	Chemokine (C-X-C motif) ligand 3	2.4	0.019
BC079460	Ccl6	Chemokine (C-C motif) ligand 6	2.2	0.002
NM_031512	Il1b	Interleukin 1 beta	2.1	0.040
NM_012758	Syk	Spleen tyrosine kinase	2.1	0.032
NM_053619	C5ar1	Complement component 5a receptor 1	2.1	0.012
NM_001007612	Ccl7	Chemokine (C-C motif) ligand 7	2.1	0.038
NM_053647	Cxcl2	Chemokine (C-X-C motif) ligand 2	2.1	0.046
NM_031512	Il1b	Interleukin 1 beta	2.0	0.046
		**Adhesion**		
NM_053572	Cdhr1	Cadherin-related family member 1	2.6	0.049
NM_001033998	Itgal	Integrin, alpha L	2.1	0.014
NM_012758	Syk	Spleen tyrosine kinase	2.1	0.032
		**Immune system**		
NM_013040	Abcc9	ATP-binding cassette, subfamily C (CFTR/MRP), member 9	2.7	0.024
NM_016994	C3	Complement component 3 Toll-like receptor 7	2.6	0.001
NM_001097582	Tlr7	Potassium inwardly-rectifying channel, subfamily J, member 8	2.5	0.002
NM_017099	Kcnj8	Fc fragment of IgG, high affinity Ia, receptor (CD64)	2.4	0.009
NM_001100836	Fcgr1a	Complement factor H	2.2	0.008
NM_130409	Cfh		2.1	0.006
		**Apoptosis**		
NM_001007729	Pf4	Platelet factor 4	2.7	0.004
NM_030871	Pde1a	Phosphodiesterase 1A, calmodulin-dependent	2.2	0.014
NM_001100836	Fcgr1a	Fc fragment of IgG, high affinity Ia, receptor (CD64)	2.2	0.008
NM_031512	Il1b	Interleukin 1 beta	2.1	0.040
NM_053619	C5ar1	Complement component 5a receptor 1	2.1	0.012
NM_012762	Casp1	Caspase 1	2.1	0.015
		**Secretion**		
NM_017043	Ptgs1	Prostaglandin-endoperoxide synthase 1	2.6	0.027
XM_006225973	Pram1	PML-RARA regulated adaptor molecule 1	2.2	0.008
NM_031512	Il1b	Interleukin 1 beta	2.1	0.040
NM_012758	Syk	Spleen tyrosine kinase	2.1	0.032
NM_012762	Casp1	Caspase 1	2.1	0.015
		**Taxis**		
NM_139089	Cxcl10	Chemokine (C-X-C motif) ligand 10	2.8	0.006
NM_001007729	Pf4	Platelet factor 4	2.7	0.004
NM_138522	Cxcl3	Chemokine (C-X-C motif) ligand 3	2.4	0.019
BC079460	Ccl6	Chemokine (C-C motif) ligand 6	2.2	0.002
NM_031512	Il1b	Interleukin 1 beta	2.1	0.040
NM_012758	Syk	Spleen tyrosine kinase	2.1	0.032
NM_053619	C5ar1	Complement component 5a receptor 1	2.1	0.012
NM_001007612	Ccl7	Chemokine (C-C motif) ligand 7	2.1	0.038
NM_053647	Cxcl2	Chemokine (C-X-C motif) ligand 2	2.1	0.046
		**Skeletal system**		
NM_013028	Shox2	short stature homeobox 2	3.3	0.022
NM_012921	Alx1	ALX homeobox 1	2.0	0.049
		**Regionalization**		
XM_006257585	Zic3	Zic family member 3	7.5	0.012
NM_012921	Alx1	ALX homeobox 1	2.0	0.049
		**Regulation of cellular localization**		
NM_017043	Ptgs1	Prostaglandin-endoperoxide synthase 1	2.6	0.027
XM_006225973	Pram1	PML-RARA regulated adaptor molecule 1	2.2	0.008
NM_031512	Il1b	Interleukin 1 beta	2.1	0.040
NM_012758	Syk	Spleen tyrosine kinase	2.1	0.032
NM_012762	Casp1	Caspase 1	2.1	0.015
		**Defense response**		
NM_139089	Cxcl10	Chemokine (C-X-C motif) ligand 10	2.8	0.006
NM_013040	Abcc9	ATP-binding cassette, subfamily C (CFTR/MRP), member 9	2.7	0.024
NM_016994	C3	Complement component 3	2.6	0.001
NM_001097582	Tlr7	Toll-like receptor 7	2.5	0.002
NM_017099	Kcnj8	Potassium inwardly-rectifying channel, subfamily J, member 8	2.4	0.009
NM_138522	Cxcl3	Chemokine (C-X-C motif) ligand 3	2.4	0.019
FQ217794	Cybb	Cytochrome b-245, beta polypeptide	2.3	0.038
NM_001100836	Fcgr1a	Fc fragment of IgG, high affinity Ia, receptor (CD64)	2.2	0.008
NM_031512	Il1b	Interleukin 1 beta	2.1	0.040
NM_130409	Cfh	Complement factor H	2.1	0.006
NM_053619	C5ar1	Complement component 5a receptor 1	2.1	0.012
NM_001007612	Ccl7	Chemokine (C-C motif) ligand 7	2.1	0.038
NM_053647	Cxcl2	Chemokine (C-X-C motif) ligand 2	2.1	0.046
NM_198769	Tlr2	Toll-like receptor 2	2.0	0.003
		**Protein maturation**		
NM_016994	C3	Complement component 3	2.6	0.001
NM_130409	Cfh	Complement factor H	2.1	0.006
NM_012762	Casp1	Caspase 1	2.1	0.015


**Table 2:** Functional classification of upregulated genes in motor fibroblasts.

Genbank accession	Gene symbol	Gene name	Fold change	*P*-values
		**Adhesion**		
NM_153621	Dab1	Dab, reelin signal transducer, homolog 1 (Drosophila)	0.5	0.007
NM_017242	Lsamp	Limbic system-associated membrane protein	0.5	0.008
XM_006232194	Nlgn1	Neuroligin 1	0.5	0.015
NM_031069	Nell1	NEL-like 1 (chicken)	0.4	0.002
NM_012929	Col2a1	Collagen, type II, alpha 1	0.3	0.023
		**Neuron differentiation**		
XM_006232194	Nlgn1	Neuroligin 1	0.5	0.015
NM_053601	Nnat	Neuronatin	0.4	0.003
NM_031737	Nkx6-1	NK6 homeobox 1	0.3	1 × 10^-4^
		**Secretion**		
NM_053601	Nnat	Neuronatin	0.4	0.003
NM_031737	Nkx6-1	NK6 homeobox 1	0.3	1 × 10^-4^
		**Sketetal system**		
NM_001007012	Alx3	ALX homeobox 3	0.4	0.036
NM_012929	Col2a1	Collagen, type II, alpha 1	0.3	0.023
NM_001107787	Pax1	Paired box 1	0.2	0.002
		**Apoptosis/Cell death**		
NM_012999	Pcsk6	Proprotein convertase subtilisin/kexin type 6	0.5	0.000
NM_001007012	Alx3	ALX homeobox 3	0.4	0.036
NM_031069	Nell1	NEL-like 1 (chicken)	0.4	0.002
NM_012929	Col2a1	Collagen, type II, alpha 1	0.3	0.023
		**Embryonic development**		
NM_001007012	Alx3	ALX homeobox 3	0.4	0.036
NM_012929	Col2a1	Collagen, type II, alpha 1	0.3	0.023
NM_001107787	Pax1	Paired box 1	0.2	0.002
		**Segmentation**		
NM_012999	Pcsk6	Proprotein convertase subtilisin/kexin type 6	0.5	1 × 10^-4^
NM_001107787	Pax1	Paired box 1	0.2	0.002
		**Regionalization**		
NM_012999	Pcsk6	Proprotein convertase subtilisin/kexin type 6	0.5	1 × 10^-4^
NM_031737	Nkx6-1	NK6 homeobox 1	0.3	1 × 10^-4^
NM_001107787	Pax1	Paired box 1	0.2	0.002
		**Pattern specification process**		
NM_012999	Pcsk6	Proprotein convertase subtilisin/kexin type 6	0.5	1 × 10^-4^
NM_001007012	Alx3	ALX homeobox 3	0.4	0.036
NM_031737	Nkx6-1	NK6 homeobox 1	0.3	1 × 10^-4^
NM_001107787	Pax1	Paired box 1	0.2	0.002


### Validation of Differentially Expressed Genes by qPCR

The genome microarray analysis showed that 16 differentially expressed genes in sensory Fbs were related to proliferation and migration that play a critical role in nerve regeneration. Several genes (*Cxcl10*, *Cxcl3*, *Abcc9*, *Syk*, *C5ar1*, *Pf4*, *Ptgs1*, and *Il1b*) involved in proliferation, migration, and chemotaxis were selected to further evaluation by qPCR. For 7 of these genes *Cxcl10*, *Cxcl3*, *Abcc9*, *Syk*, *C5ar1*, *Pf4*, and *Ptgs1*, higher expression in sensory *vs*. motor Fbs was confirmed (Figure 2C). However, there were no differences between motor and sensory Fbs for expression of *Il1b*. This might be attributed to individual differences or to heterogeneity of samples.

### Cell Proliferation and Migration Analysis

**FIGURE 3:**
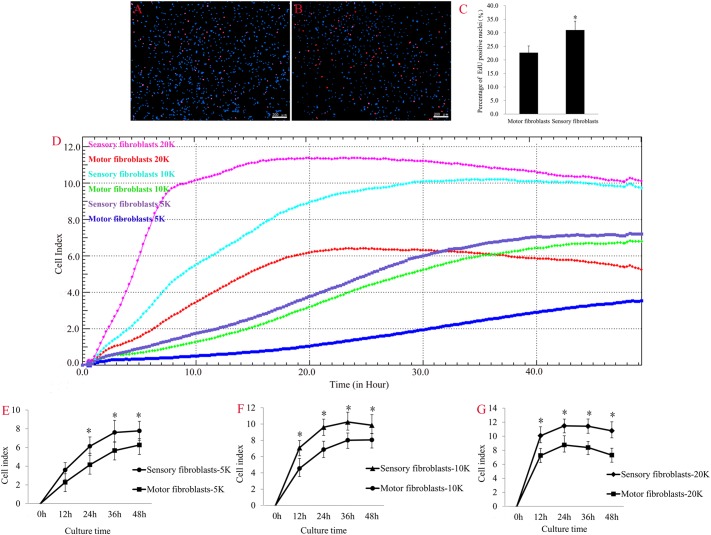
The merge of EdU staining (red) and Hoechst 33342 staining (blue) for cultured motor **(A)** and sensory **(B)** fibroblasts, in which scale bar is 200 μm. **(C)** The histogram showing the percentage of EdU-positive nuclei in motor and sensory fibroblasts. *p* = 0.041, unpaired *t*-test, n = 9 rats. **(D)** Proliferation capacity of motor and sensory fibroblasts in three different cell density of 5K, 10K, 20K continuously monitored by RTCA for 48 h. Quantification of cell proliferation index of cultured sensory and motor fibroblasts in three different cell density of 5K **(E)**, 10K **(F)**, 20K **(G)**. ^∗^*p* < 0.05 vs. motor fibroblasts, unpaired *t*-test, *n* = 14–15 rats.

Fibroblasts are activated to proliferate and migrate in nerve regeneration under pathological conditions. EdU (red)/Hoechst (blue) immunostaining showed that the number of EdU-positive Fbs was significantly higher in sensory Fbs compared to motor Fbs (Figures 3A–C). These results demonstrated a higher proliferation rate of sensory Fbs than motor Fbs. The results of RTCA showed that the rate of proliferation of sensory Fbs plated at three different densities (5K, 10K, 20K per well) was significantly more than that of motor Fbs (Figures 3D–G).

**FIGURE 4:**
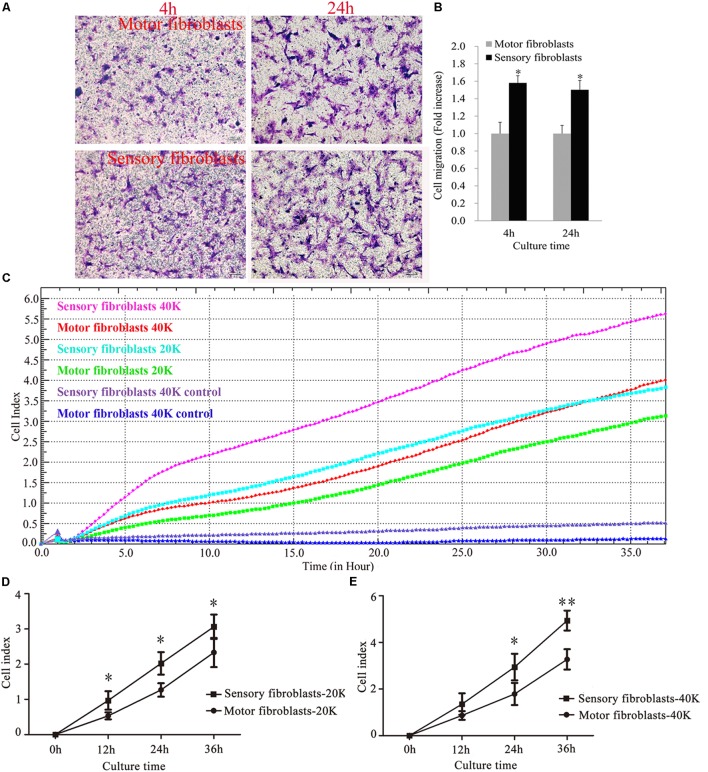
Images showing cultured sensory and motor fibroblasts migrating to the bottom surface of the trans-well membrane after 4 or 24h **(A)**. Scale bar, 100 μm. **(B)** The histogram demonstrating the quantitative analysis in cell migration between cultured sensory and motor fibroblasts after 4 or 24 h. *p* = 0.0016 and *p* = 0.0011, respectively, unpaired *t*-test, *n* = 18–19 rats. Migration capacity of motor and sensory fibroblasts in different cell density of 20K, 40K continuously monitored by RTCA for 36 h **(C)**. Quantification of cell migration index of cultured motor and sensory fibroblasts in two different cell density of 20K **(D)**, 40K **(E)**. ^∗^*p* < 0.05, ^∗∗^*p* < 0.01 vs. motor fibroblasts, unpaired *t*-test, *n* = 18 rats.

The transwell assay showed that after migration for 4 h or 24 h, the rate of migration of sensory Fbs was higher than that of motor Fbs (Figures 4A,B). The results of RTCA showed that the migration rate of sensory Fbs plated at different densities (20K, 40K) was significantly faster than that of motor Fbs (Figures 4C–E).

### Involvement of *Cxcl10* and *Cxcl3* in the Proliferation and Migration of Fbs

**FIGURE 5:**
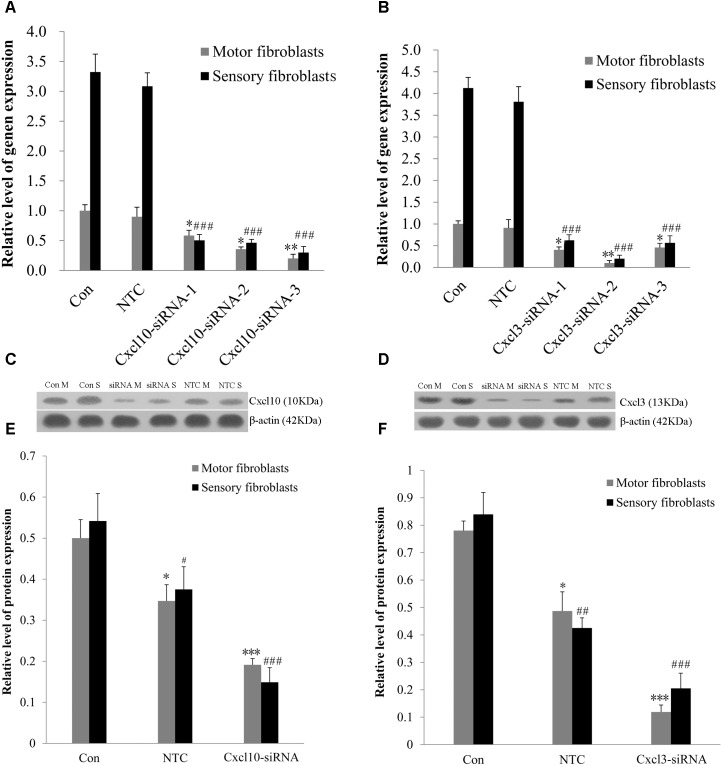
**(A,B)** The knockdown efficiency of *Cxcl10* and *Cxcl3*-targeting siRNAs was detected in primary motor and sensory fibroblasts, three siRNAs for each target. Motor and sensory fibroblasts were transfected with NTC siRNA or with *Cxcl10* and *Cxcl3*-specific siRNA, their mRNA levels were detected by qPCR after transfection for 48 h. ^∗^*p* < 0.05, ^∗∗^*p* < 0.01 vs. motor fibroblasts Con, ^###^*p* < 0.001 vs. sensory fibroblasts Con, *n* = 9 rats. **(C,D)** Motor and sensory fibroblasts were transfected with NTC siRNA or with *Cxcl10* and *Cxcl3*-specific siRNA for 48 h. The fibroblasts extracts were subjected to Western blot to test the levels of these endogenous proteins; β-actin served as an internal control. Quantification of protein expression in motor and sensory fibroblasts after transfection with NTC siRNA or with *Cxcl10* and *Cxcl3*-specific siRNA for 48 h **(E,F)**. Data are presented as means ± SEM. ^∗^*p* < 0.05, ^∗∗∗^*p* < 0.001 compared with Motor fibroblasts Con, ^#^*p* < 0.05, ^##^*p* < 0.01, ^###^*p* < 0.001 compared with Sensory fibroblasts Con, *n* = 9 rats.

The genome microarray analysis identified the genes related to cell proliferation and migration, such as chemokine (C-X-C motif) ligand 10 (*CXCL10*) and chemokine (C-X-C motif) ligand 3 (*CXCL3*). The gene expression levels in sensory Fbs were 2.8- and 2.4-fold, respectively, higher than that in motor Fbs (Table 1). In order to elucidate the role of these genes in the proliferation and migration of Fbs, siRNA transfection was used to selectively suppress their expression in motor and sensory Fbs. Transfection using NTC siRNA did not alter the expression of *CXCL10* or *CXCL3* mRNA. However, the expression was reduced by >70% by CXCL10 or CXCL3-selective siRNA (Figures 5A,B). The most effective siRNA for each chemokine was 3# CXCL10 siRNA and 2# CXCL3 siRNA, respectively, which was used in the following experiments. Subsequently, Western blot results demonstrated the efficient knockdown of both by both siRNAs (Figures 5C–F). These data showed the expression of CXCL10 and CXCL3 was significantly suppressed by siRNA transfection.

**FIGURE 6:**
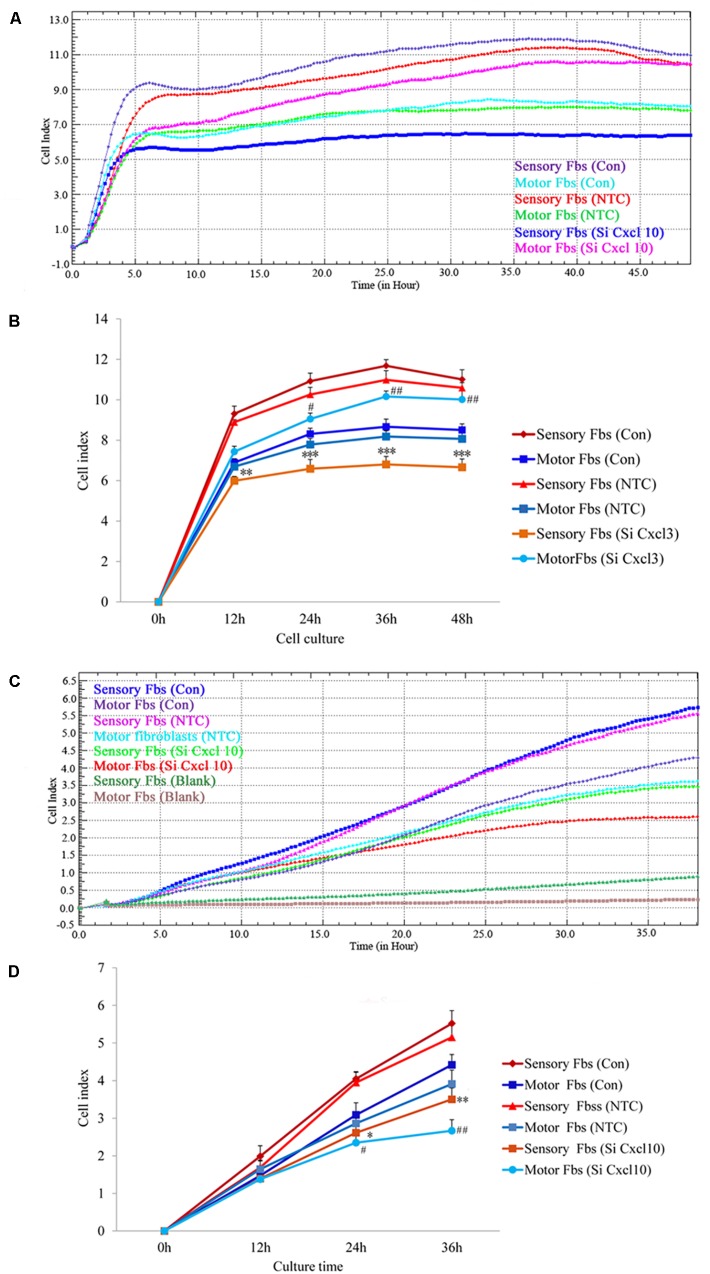
Proliferation **(A)** and migration **(C)** capacity of motor and sensory fibroblasts after transfection with NTC siRNA or with Cxcl10-specific siRNA for 48 h continuously monitored by RTCA for 48 or 36 h. Quantification of cell proliferation **(B)** and migration **(D)** index of cultured motor and sensory fibroblasts after transfection with NTC siRNA or with Cxcl10-specific siRNA for 48 h. ^∗^*p* < 0.05, ^∗∗^*p* < 0.01, ^∗∗∗^*p* < 0.001 compared with Sensory Fbs Con, ^#^*p* < 0.05, ^##^*p* < 0.01, compared with Motor Fbs Con, *n* = 21 rats for cell proliferation assay, *n* = 24 rats for cell migration assay.

**FIGURE 7:**
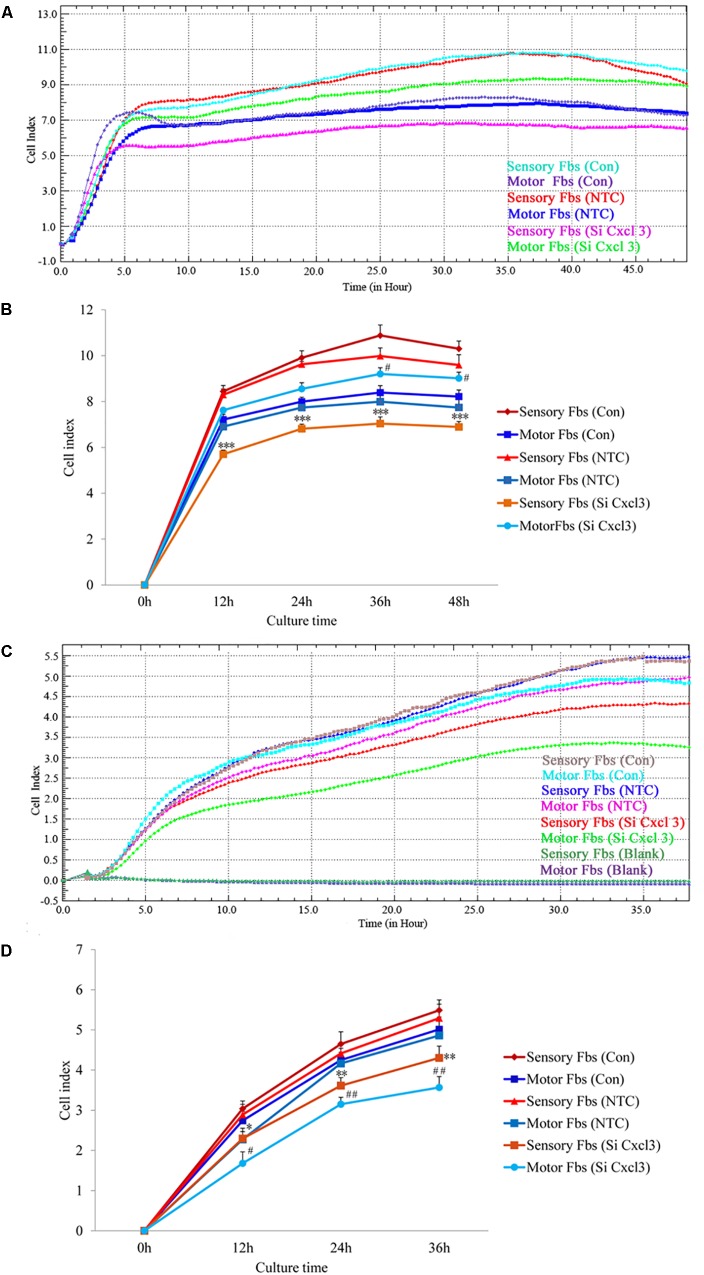
Proliferation **(A)** and migration **(C)** capacity of motor and sensory fibroblasts after transfection with NTC siRNA or with Cxcl3-specific siRNA for 48 h continuously monitored by RTCA for 48 or 36 h. Quantification of cell proliferation **(B)** and migration **(D)** index of cultured motor and sensory fibroblasts after transfection with NTC siRNA or with Cxcl3-specific siRNA for 48 h. ^∗^*p* < 0.05, ^∗∗^*p* < 0.01, ^∗∗∗^*p* < 0.001 compared with Sensory Fbs Con, ^#^*p* < 0.05, ^##^*p* < 0.01, compared with Motor Fbs Con, *n* = 21 rats for cell proliferation assay, *n* = 28 rats for cell migration assay.

The effect of CXCL10 and CXCL3 on the proliferation and migration of Fbs was detected by RTCA assay. No significant differences were observed between control and NTC. Compared to the control, the rate of proliferation of sensory Fbs (10K) was significantly suppressed by CXCL10-siRNA infection, while the proliferation rate of motor Fbs (10K) was significantly enhanced by CXCL10-siRNA infection (Figures 6A,B). However, the rate of migration of sensory and motor Fbs was suppressed by CXCL10-siRNA infection (Figures 6C,D). Similar to CXCL10, the proliferation rate of sensory Fbs (10K) was significantly suppressed by CXCL3-siRNA infection, while the proliferation rate of motor Fbs (10K) was enhanced by CXCL 3-siRNA infection (Figures 7A,B). However, the migration rate of sensory and motor Fbs was suppressed by CXCL3-siRNA infection (Figures 7C,D). To the best of our knowledge, this was the first study that demonstrated the involvement of CXCL10 and CXCL3 in the proliferation and migration of peripheral nerve Fbs.

### Motor and Sensory Fbs/SC Co-culture Assay

**FIGURE 8:**
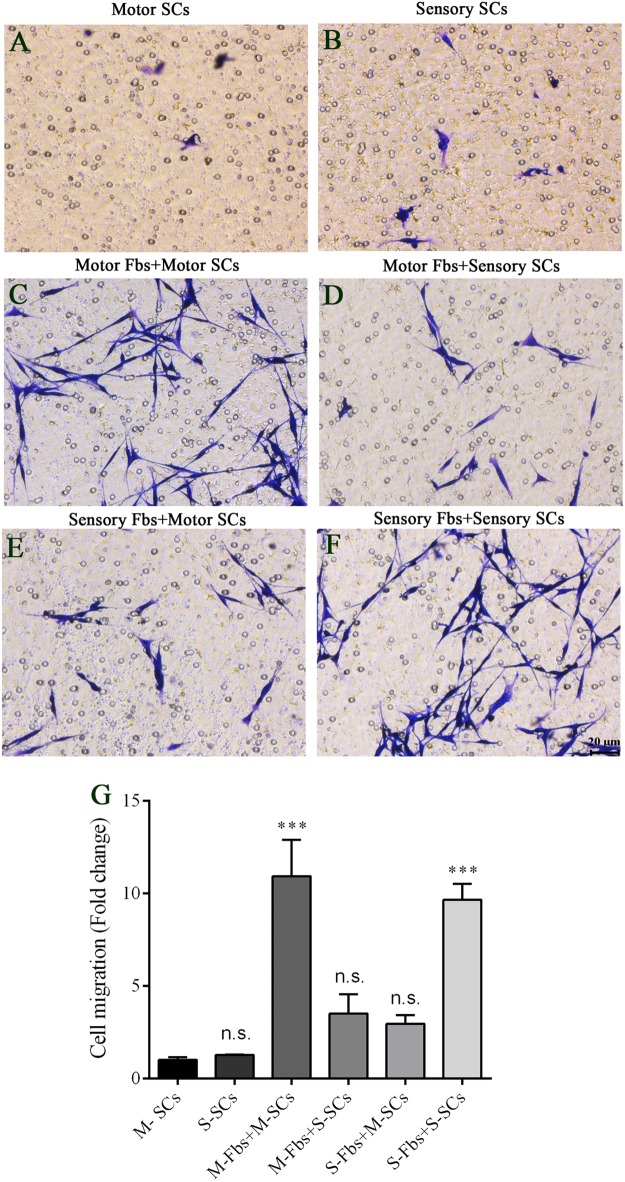
**(A–F)** Images showing motor and sensory SCs and motor and sensory SCs co-cultured with motor and sensory Fbs migrating to the bottom surface of the trans-well membrane after co-cultured for 12 h. Scale bar, 20 μm. **(G)** Quantification of SCs migrating to the bottom surface of the trans-well membrane. *p* = 0.0001 and *p* = 0.0005, respectively, ^∗∗∗^*p* < 0.001 compared with M-SCs group, one-way ANOVA, *n* = 18–19 rats.

The co-culture experiments were designed to detect the effects of different phenotypic Fbs on the migration of SCs. The co-culture assay showed that when the motor or sensory SCs were plated alone, only a few SCs migrated to the bottom of the insert membrane. However, the cell number significantly increased in motor SCs co-culturedx with motor Fbs group and sensory SCs co-cultured with sensory Fbs group. But only a low number of SCs migrated to the bottom of the insert membrane in sensory SCs co-cultured with the motor Fbs group or motor SCs co-cultured with the sensory Fbs group (Figure 8).

## Discussion

### Peripheral Nerve Fibroblasts Express Distinct Sensory and Motor Phenotypes

Fibroblasts are present in all tissues and play a key role in the synthesis and secretion of an extracellular matrix (ECM), wound healing, angiogenesis, locomotion, collagen, and elastin fiber production (Sorrell and Caplan, 2009; Bautista-Hernandez et al., 2017). As a response to severe peripheral nerve injury, a large number of Fbs accumulate at the injury site in the early stages of injury, and then, axon growth is guided by regulating the sorting and migration of SCs via ephrin-B/EphB2 signaling pathway (Parrinello et al., 2010). Numerous studies have indicated that after peripheral nerve injury, sensory and motor nerve regeneration pathways are different. The transected femoral motor axons preferentially re-innervated muscle branch rather than a cutaneous branch, a process that is termed “preferential motor reinnervation” (PMR) (Brushart, 1988). PMR was also verified when access to target muscle was denied (Brushart, 1993). These studies strongly suggested that SCs and Fbs tubes can specifically identify the motor and sensory axons and influence their regeneration. Reportedly, SCs express distinct motor and sensory phenotypes and neurotrophic factors to selectively promote the motor and sensory axon growth (Hoke et al., 2006; Allodi et al., 2012; Jesuraj et al., 2012). Whether peripheral nerve Fbs also express distinct motor and sensory phenotypes remains to be defined.

In this study, we compared the gene expression patterns of motor and sensory Fbs using the whole rat genome microarray analysis and identified a total of 121 genes differentially expressed between the primary cultured motor and sensory Fbs. The differentially expressed genes with a high expression level in sensory Fbs were involved in biological aspects such as proliferation, migration, chemotaxis, and motility activation. The marked difference in the expression level of some key genes, including *Cxcl10*, *Cxcl3*, *Abcc9*, *Syk*, *C5ar1*, *Pf4*, and *Ptgs1*, involved in proliferation and migration, was validated by qPCR. A previous study showed that the high expression of *Abcc9* gene was significantly associated with the proliferation of epithelial ovarian cancer cells (Elsnerova et al., 2017). C5aR1, a receptor for complement component 5a, promotes proliferation and polarization of embryonic neural progenitor cells through protein kinase C ζ signaling pathway (Coulthard and Hawksworth, 2017). Moreover, C5aR1 also promotes bone metastasis of lung cancer by CXCL16-mediated effects (Ajona et al., 2018). The colony formation, migration, and proliferation of human and murine glioma samples and cell lines were effectively blocked through SYK inhibition (Moncayo et al., 2018). In order to examine whether these highly expressed migration- and proliferation-related genes in sensory Fbs affected the biological behavior of the Fbs, we performed EdU assay, real-time cell analysis, and transwell migration assay to assess the migration and proliferation of sensory and motor Fbs. We found that the proliferation or migration rate of sensory Fbs was faster than that of motor Fbs in a cell density-dependent manner. These results indicated that the high expression genes associated with migration and proliferation in sensory Fbs contribute toward the altered cell behavior. Furthermore, the Fbs express distinct motor and sensory phenotypes, which are associated with the differential expression of the genes and biological processes. However, whether the Fbs with distinct phenotypes play a role in neural development and regeneration process is yet to be elucidated.

The Fbs and SCs co-culture assay showed that motor Fbs had a high affinity for motor SCs than for sensory SCs, and vice versa. Also, the identical subtype displayed a higher affinity of Fbs and SCs for each other. Because the Fbs and SCs are separated by the transwell membrane, it is likely that soluble signaling factors are secreted by the Fbs. Previous studies have shown that in the early stage after nerve transection, a large number of Fbs accumulate at the site of injury, and then, regulate the sorting and migration of SCs as well as the axon growth (Parrinello et al., 2010). Thus, we speculated that after peripheral nerve injury, motor and sensory Fbs attract SCs with the same phenotype to form different bands of Büngner, which can guide the corresponding motor or sensory axons into the accurate motor or sensory regeneration pathway. Thus, understanding the molecular interaction between motor and sensory axons, SCs, and Fbs, and developing accurate therapies is essential to promote the functional recovery of peripheral axon regeneration. The current study demonstrated that Fbs express distinct motor and sensory phenotypes that are associated with differential gene expression, biological processes, and effects on SCs. However, whether different proliferation and migration rates of Fbs affect the function or regeneration rate of sensory and motor nerves and whether the different phenotypes of Fbs affect the peripheral nerve development and regeneration necessitates further exploration.

### Role of CXCL10 and CXCL3 in Proliferation/Migration of Sensory and Motor Fbs

Chemokines are a family of small cytokines or signaling proteins that are secreted locally in cells and are specialized in attracting inflammatory and structural cells to the sites of injury (Luo et al., 2016; Zhang et al., 2017). These enzymes have been shown to regulate the proliferation and migration in many cell types (de Haas et al., 2007). CXCL10, a member of chemokine family, mediates a series of biological activities, including migration, chemotaxis, growth, apoptosis, angiogenesis, and neuropathic pain by binding with the CXCR3 receptor (Ejaeidi et al., 2015; Sanchez-Lugo et al., 2015; Jiang et al., 2017). In murine habu nephritis, the proliferation of mesangial cells was inhibited via ERK signaling pathway by the knockdown of *CXCL10* gene (Gao et al., 2017). However, the treatment with CXCL10 significantly reduced the *in vitro* invasiveness of melanoma, as well as reduced the melanoma tumor growth and metastasis in C57BL/6 mice (Antonicelli et al., 2011). CXCL3 also belongs to the chemokine subfamily, and is widely expressed and involved in tissue damage and inflammatory responses (Zhang L. et al., 2016). A previous study showed that mesenchymal stem cells inhibited T cell proliferation via CXCL3; the molecule binds with CXCR2 receptor on the surface of T cells to suppress the activation of AKT, signal transducer, and activator of transcription (STAT) 3 and Janus kinase (JAK) 2 (Lee Y. S. et al., 2012). The current results showed that knockdown of CXCL10 or CXCL3 significantly suppressed the proliferation rate of sensory Fbs, while enhanced that of motor Fbs. In human cells, the receptor of CXCL10, CXCR3 includes three isoforms: CXCR3-A, CXCR3-B, and CXCR3-alt, which are linked to alternative splicing and distinct functions (Liu et al., 2011; Billottet et al., 2013). Furthermore, CXCR3-A is related to the pro-survival functions, whereas CXCR3-B mediates suppressive effects on cell migration and proliferation; however, these different effects of the CXCL10 receptor are cell- and stimuli-specific (Wu et al., 2012; Utsumi et al., 2014). In this study, CXCL10 might effectuate as a double-edged sword in the regulation of proliferation of sensory and motor Fbs. Thus, we speculated that both CXCL10 and CXCL3 might regulate the cell cycle of sensory and motor Fbs through binding to different receptors and stimulating various downstream signaling pathways. However, the mechanism underlying the CXCL10- and CXCL3-regulated proliferation of sensory and motor Fb needs further exploration.

Structural cell migration is a critical phenomenon involved in various physiological and pathophysiological conditions. CXCL10 has been demonstrated to exert a positive effect on tumor cell invasion, migration, and survival in diverse human tumor cell lines (Zipin-Roitman et al., 2007; Lee J. H. et al., 2012; Tokunaga et al., 2018). The regular time-dependent expression of CXCL10, CCL2, CCL3, and CCL5 suggests a chemoattractant effect on inflammatory cell migration, angiogenesis, and nerve regeneration during tendon repair (Stalman et al., 2015). CXCL10 also mediated an *in vitro* hippocampal astrocyte migration in Alzheimer’s disease (Lai et al., 2013). The overexpression of CXCL3 and its receptor CXCR2 promoted the proliferation and migration of cancer cells by regulating the expression of genes, including *ERK*, *BAX*, *TP73*, and NDRG3, which enhance the oncogenic potential of prostate cancer (Gui et al., 2016). Furthermore, CXCL3-induced migration of asthmatic airway smooth muscle cells was dependent on p38 and ERK1/2 MAPK signaling pathways (Al-Alwan et al., 2013). In the current study, the role of CXCL10 and CXCL3 in the migration of Fbs was assessed by knockdown experiments. The rate of migration of sensory and motor Fbs was suppressed by CXCL10- or CXCL3-siRNA infection. This demonstrated that CXCL10 and CXCL3 exerted a positive effect in modulating the migration of sensory and motor Fbs. However, the mechanism underlying the signaling pathway of CXCL10-regulated migration of peripheral nerve sensory and motor Fbs is yet to be explored. To the best of our knowledge, this was the first study demonstrating the involvement of CXCL10 and CXCL3 in the proliferation and migration of peripheral nerve Fbs.

## Conclusion

Whole rat genome microarray was used to analyze the differentially expressed genes in primary cultured sensory and motor Fbs and to identify 121 differentially expressed genes between both Fbs. The high expression genes in the sensory Fbs were involved in the biological aspects such as proliferation, migration, chemotaxis, motility activation, protein maturation, defense response, immune system, taxis, and regionalization, while those in motor Fbs were involved in neuron differentiation, segmentation, and pattern specification process. Furthermore, the rate of cell migration or proliferation was higher in sensory Fbs than in motor Fbs. The Fbs and SCs co-culture assay showed that motor Fbs appeared more attractive to motor SCs than to sensory SCs, and sensory Fbs were more attractive to sensory SCs than to motor SCs. Moreover, the downregulated expression of CXCL10 or CXCL3 suppressed the rate of proliferation of sensory Fbs and enhanced that of motor Fbs. However, the rate of migration of sensory and motor Fbs was suppressed by downregulated expression of CXCL10 or CXCL3. Thus, Fbs express distinct motor and sensory phenotypes that are associated with differential gene expression, biological process, and effects on SCs. These findings might aid in elucidating the biological differences between motor and sensory Fbs, including the role in peripheral nerve regeneration.

## Author Contributions

QH, MS, FD, and YG conceived and designed the experiments. QH, MS, and FT performed the experiments. QH and MS analyzed the data. MC and SZ contributed to reagents, materials, and analysis tools. QH, FD, and YG wrote the manuscript. All authors have read and approved the final manuscript.

## Conflict of Interest Statement

The authors declare that the research was conducted in the absence of any commercial or financial relationships that could be construed as a potential conflict of interest.

## Supplementary Material

Table S1 Primer sequences for qPCR.

Table S2 Sequences of siRNA.

Table S3 The original normalized data for whole rat genome microarray analysis.

Table S4 121 differential expressed genes in motor and sensory Fbs.
