# Supplementary material for: Differential Gene Expression in Primary Cultured Sensory and Motor Nerve Fibroblasts
Source: Front Neurosci. 2019 Jan 9;12:1016. doi: 10.3389/fnins.2018.01016 (PMC6333708; doi:10.3389/fnins.2018.01016)
Supplement: Table S1 — Primer sequences for qPCR. [file Table_1.DOCX]

**Table 1** Primer sequences for qPCR

| Gene name | NCBI Reference Sequence | Gene sequence ( 5’ to 3’) |
| --- | --- | --- |
| Gapdh | NM_017008.4 | AAGTTCAACGGCACAGTCAAG  CCAGTAGACTCCACGACATACTCA |
| Cxcl10 | NM_139089 | ATTGAAAGCGGTGAGCCAAAGAA  ACTGGGTAAAGGGAGGTGGAGA |
| Ptgs1 | NM_017043 | AAGGGAAGAAGCAGTTACCAGATAT  GCAAAGAAAGCAAACAAGACGT |
| Abcc9 | NM_013040 | AACCGAAGAATAACACGACGAGA  TGTAGATCATAGCCAGCAGAGCC |
| Syk | NM_012758 | TACGGGCAGAAGCCATACAGAG  AAGTCCAGCATAGGAACATCAAGTC |
| Cxcl3 | NM_138522 | GCACCCAGACAGAAGTCATAGCC  CAAGGGATCGACTCGGACGTTAT |
| Pf4 | NM_001007729 | GCTTCTTCTGGGTCTGCTGTTGC  CAGGCTGGTGATGCGTTTGAGAT |
| C5ar1 | NM_053619 | GCGGAGTAACCTGGGTCTTAGCATT  AGAGTGAGCAGAGGCAACACGAAAC |
