# Supplementary material for: Differential Gene Expression in Primary Cultured Sensory and Motor Nerve Fibroblasts
Source: Front Neurosci. 2019 Jan 9;12:1016. doi: 10.3389/fnins.2018.01016 (PMC6333708; doi:10.3389/fnins.2018.01016)
Supplement: Table S2 — Sequences of siRNA. [file Table_2.DOCX]

**Table 2** Sequences of siRNA

| **Gene name** | **Target sequence** |
| --- | --- |
| si-r-Cxcl10_001 | GAGTCTGAGTGGGACTCAA |
| si-r-Cxcl10_002 | GCATCGACTTCCATGAACA |
| si-r-Cxcl10_003 | TCCGCATGTTGAGATCATT |
| si-r-Cxcl3_001 | CCTCAATGCTGCACTGCTT |
| si-r-Cxcl3_002 | GAAGACCCTACCAAGGGTT |
| si-r-Cxcl3_003 | GCACCCAGACAGAAGTCAT |
